# T-Cell-Based Universal Dengue Vaccine Design for Robust Protective Response

**DOI:** 10.3390/vaccines13111118

**Published:** 2025-10-30

**Authors:** Yi Fei Sun, Adeline Yeo Syin Lian, Meng Ling Moi

**Affiliations:** Department of Developmental Medical Sciences, Graduate School of Medicine, The University of Tokyo, Tokyo 113-0033, Japan; sun-yifei708@g.ecc.u-tokyo.ac.jp (Y.F.S.); adelineyeo@m.u-tokyo.ac.jp (A.Y.S.L.)

**Keywords:** dengue fever, T-cell epitope-based vaccine, antibody-dependent enhancement, serotype diversity, epitope prediction, next-generation platforms

## Abstract

Dengue virus remains a major global health threat due to the lack of a safe and broadly effective vaccine. Traditional antibody-based vaccines often show limited protection and can exacerbate disease severity in individuals without prior exposure. A new generation of T-cell epitope-based vaccines offers a promising and safer approach by activating the cellular arm of the immune system to complement antibody responses. Instead of targeting only surface structural proteins, these vaccines focus on highly conserved peptide regions within non-structural proteins, particularly NS3 and NS5, that are shared across all four dengue virus serotypes. Peptides such as DTTPFGQQR, KPGTSGSPI, and MYFHRRDLRL have been identified as potent immunogenic targets capable of inducing strong cytotoxic and helper T-cell responses, promoting viral clearance and long-term immune memory. Advanced immunoinformatic enables precise prediction and selection of epitopes with high binding affinity to human leukocyte antigens and broad cross-serotype conservation. These peptides can be integrated into next-generation vaccine delivery systems, including messenger RNA and nanoparticle platforms, which enhance antigen presentation, improve molecular stability, and reduce the risk of antibody-dependent disease enhancement. Together, this integrative design represents a rational path toward a safer, cross-protective, and durable dengue vaccine that closely mimics the balanced cellular and humoral immunity observed after natural infection, offering renewed hope for effective global dengue prevention.

## 1. Introduction

Dengue fever (DF) represents a formidable and rapidly expanding major public health issue, particularly prevalent in tropical and subtropical regions across the globe. This debilitating disease is caused by the dengue virus (DENV), a single-stranded, positive-sense RNA virus that belongs to the Flaviviridae family and the Flavivirus genus. The DENV genome encodes for a total of ten proteins. Three of these are structural proteins: the capsid (C), pre-membrane (prM), and envelope (E) proteins. These structural components are essential for the assembly, integrity, and morphology of the viral particle, playing a critical role in how the virus is shaped and structured. The remaining seven proteins are non-structural proteins: NS1, NS2A, NS2B, NS3, NS4A, NS4B, and NS5. These non-structural proteins are indispensable for various stages of the viral life cycle, including viral replication, and are crucial for the virus’s ability to evade and counteract the host immune system [1].

The primary mode of DENV transmission to humans is through the bite of infected mosquitoes, predominantly *Aedes aegypti* and, to a lesser extent, *Aedes albopictus* [2]. The escalating impact of climate change has significantly exacerbated the global burden of dengue. Warmer global temperatures facilitate the expansion of mosquito habitats into previously unaffected geographical areas and prolong the breeding seasons of these vectors, consequently increasing the population at risk of infection [3]. There are four genetically distinct serotypes of DENV: DENV-1, DENV-2, DENV-3, and DENV-4. An individual can be infected by any of these serotypes. However, a critical characteristic of dengue immunity is that infection with one serotype confers lifelong protection only against that specific serotype. It does not provide long-term, cross-protective immunity against the other serotypes [1], leaving individuals susceptible to subsequent infections with different DENV strains.

The clinical presentation of dengue infection is remarkably diverse, ranging from entirely asymptomatic cases to mild, self-limiting flu-like illnesses. However, in a subset of infected individuals, the disease can progress to severe and life-threatening manifestations, including dengue hemorrhagic fever (DHF) or dengue shock syndrome (DSS) [2,4]. These severe complications are characterized by internal bleeding, significant plasma leakage from blood vessels, and a drastic drop in platelet count [1,3], which can lead to hypovolemic shock and organ failure. Globally, severe dengue is responsible for over 20,000 deaths annually, with a disproportionate impact on children, who are particularly vulnerable [3,5]. A key pathological mechanism contributing to the severity of dengue is antibody-dependent enhancement (ADE) [6], together with an unbalanced T-cell response, hypothesized as the “original antigenic sin”. This ADE phenomenon occurs when non-neutralizing or sub-neutralizing antibodies, from a previous DENV infection with a different serotype or flavivirus, bind to the virus and facilitate its entry into Fcγ receptor-bearing (FcγR) cells, leading to increased viral replication and a more severe disease outcome. The interplay between Zika virus and dengue virus provides a representative case, illustrating how cross-reactive flavivirus immunity can modulate disease outcomes in either protective or pathogenic directions. Zika and dengue share enough structural similarity that antibodies raised against one virus often bind the other [7,8]. When cross-reactive antibodies are broadly neutralizing and present at high titers, they confer protection. However, at low or sub-neutralizing concentrations—such as those observed in Zika-immune macaques challenged with dengue virus or in human convalescent sera—IgG1 antibodies can bind dengue virions without neutralizing them, thereby promoting ADE that leads to increased viremia and inflammatory responses [9,10,11,12]. Maternal Zika antibodies illustrate this risk: they bind dengue but do not neutralize, leading to severe disease in offspring [13,14,15,16]. By contrast, pre-existing dengue immunity can either exacerbate Zika infection during pregnancy or reduce viremia and prevent enhancement in nonpregnant hosts [17]. Overall, these studies show that the outcome of flavivirus cross-immunity hinges on antibody quality, concentration, and infection sequence, underscoring the need for vaccines that induce durable, cross-neutralizing responses and avoid sub neutralizing antibody levels that could enhance infection.

Currently, there is no approved specific antiviral treatment available for dengue. Medical management is primarily supportive, focusing on alleviating symptoms, maintaining fluid balance, and managing potential complications such as hemorrhage or shock [1]. This lack of specific therapeutics places a heavy and often unsustainable strain on healthcare systems, particularly in endemic regions with limited resources [3]. Given these multifaceted challenges, there is an urgent and critical need for the development of a safe, effective, and broadly protective dengue vaccine. Numerous vaccine candidates are presently undergoing rigorous testing in various phases of clinical trials [18], exploring diverse platforms and approaches. This comprehensive review aims to describe on the potential of enhancing traditional antibody-based vaccine strategies by actively engaging other crucial arms of the immune system, specifically focusing on the roles of CD4+ and CD8+ T cells. Furthermore, the review will highlight the major challenges that continue to hamper the development of broadly effective dengue vaccines, emphasizing the need for innovative solutions to overcome these challenges.

## 2. Dengue Vaccine Development

Vaccines are widely recognized as one of the most impactful interventions in the control and prevention of viral diseases, having dramatically reduced morbidity and mortality from numerous infectious agents globally. In the context of dengue, extensive and sustained research efforts have been dedicated to developing an effective vaccine capable of providing broad and durable protection against all four DENV serotypes. These efforts have explored a diverse array of vaccine platforms, each with its unique characteristics, mechanisms of action, and associated advantages and limitations. Approaches include the development of live-attenuated chimeric viruses, which combine genetic material from DENV with a safe, attenuated viral backbone (e.g., yellow fever virus); traditional live-attenuated viruses, where DENV itself is weakened to induce an immune response without causing severe symptoms; inactivated viruses, which involve chemically or physically inactivating the virus while preserving its immunogenicity; recombinant proteins, focusing on specific viral proteins (e.g., envelope protein) produced in vitro to elicit targeted antibody responses; and more recently, mRNA vaccines, which deliver genetic instructions for host cells to produce viral proteins, thereby stimulating an immune response. Each of these vaccine types exhibits varying degrees of attenuation, elicits distinct patterns of immune response, and demonstrates different levels of effectiveness in clinical trials (Table 1).

Clinical trials have yielded varied and often complex results for different dengue vaccine formulations, reflecting the inherent challenges in developing a broadly effective vaccine against a virus with four distinct serotypes and the risk of ADE. The TAK-003 vaccine, for instance, has demonstrated encouraging overall efficacy, showing an 80.9% efficacy rate in children and adolescents aged 4 to 16 years. More importantly, it exhibited particularly strong protection against severe dengue, achieving a remarkable 95.4% efficacy rate in preventing hospitalization due to the disease [38]. This suggests a significant step forward in mitigating the most life-threatening forms of dengue. Dengvaxia has previously been approved for administration in children aged 9 to 16 in a limited number of countries [19,20]. Despite its licensure in various dengue endemic regions, Dengvaxia has presented significant limitations and safety concerns post-implementation. Its effectiveness has been observed to be notably low in younger children and, more critically, in individuals who have not been previously infected with dengue (dengue-naïve individuals). A major concern that emerged is its association with a higher risk of severe dengue in vaccinated individuals who were dengue-naïve at the time of vaccination [39,40,41]. TAK003 and other tetravalent vaccines have also been approved, but they will require further efficacy data from global, longer-term studies (Table 1). This phenomenon underscores the complex interplay between pre-existing immunity and vaccine-induced responses, particularly in the context of ADE.

Recent advancement in vaccine deployment for RNA viruses as shown in COVID-19 mRNA vaccines demonstrates potential of advanced vaccine platforms. The two-dose regimens of BNT162b2 (Pfizer-BioNTech, Mainz, Germany) and mRNA-1273 (Moderna, Cambridge, Massachusetts, USA) against SARS-CoV-2 exhibited exceptionally strong efficacy, providing 95% and 94.1% protection against symptomatic COVID-19, respectively [42,43]. These groundbreaking results unequivocally highlight the immense potential for developing highly effective vaccines against RNA viruses when leveraging cutting-edge platforms such as mRNA technology. This striking comparison between the outcomes of dengue and COVID-19 vaccine development underscores the need to re-evaluate and improve current dengue vaccine strategies. Lessons learned from the rapid and successful development of COVID-19 vaccines, particularly regarding platform technologies, antigen design, and rapid clinical translation, can and should guide better strategies for creating safer, more effective, and broadly protective dengue vaccines in the future, ultimately aiming to overcome the persistent global health burden of this disease.

## 3. T Cell Responses and Protection in Dengue Virus Infection

DENV-specific T cell responses are multifaceted, involving complex interplay between both CD8+ and CD4+ T cell subsets. Both CD8+ and CD4+ T cells possess the capacity to develop potent cytotoxic capabilities. This is evidenced by their ability to produce and release key effector molecules such as granzyme B and perforin, which are essential for inducing apoptosis (programmed cell death) in virus-infected cells, thereby eliminating viral factories. Specifically, CD8+ T cells primarily exert their cytotoxic functions through the recognition of viral peptides presented on MHC class I molecules, which are expressed on nearly all nucleated cells [44]. In contrast, CD4+ T cells engage with viral peptides presented on MHC class II molecules, typically found on professional antigen-presenting cells like dendritic cells, macrophages, and B cells.

Th1 cells, a crucial subset of CD4+ T cells, play a pivotal role in anti-DENV immunity. They achieve this by producing and secreting a repertoire of inflammatory cytokines, most notably interferon-gamma (IFN-γ) and tumor necrosis factor-alpha (TNF-α). These cytokines are instrumental in orchestrating and supporting the host’s antiviral defense mechanisms, including enhancing the killing capacity of other immune cells and inhibiting viral replication. Conversely, regulatory CD4+ T cells (Treg) serve a vital function in modulating and dampening excessive inflammation. They achieve this by releasing immunosuppressive cytokines such as interleukin-10 (IL-10) and transforming growth factor-beta (TGF-β), which help to maintain immune homeostasis and prevent immunopathology. T follicular helper (Tfh) cells are another indispensable subset of CD4+ T cells. They provide crucial and direct support to B cells within the specialized microenvironments of germinal centers in lymphoid organs. This support is essential for facilitating the production of high-affinity antibodies and for the formation of memory B cells and long-lived plasma cells, which are fundamental components for generating robust and enduring antibody responses that provide long-term protection [45,46].

**Figure 1:**
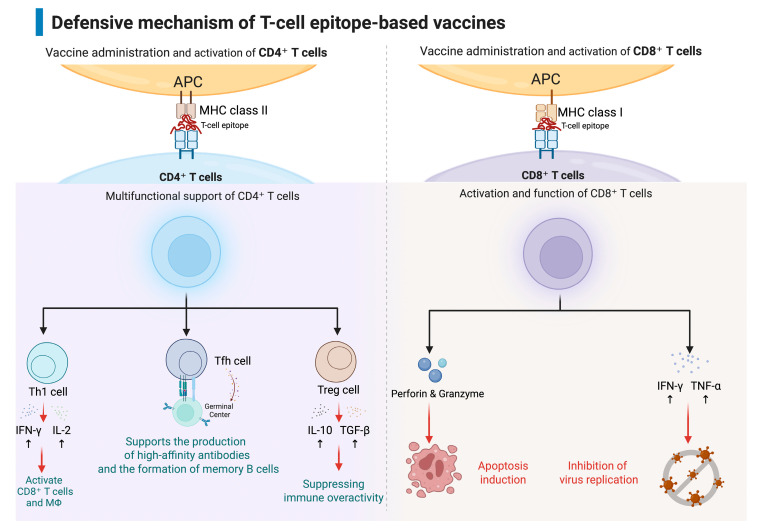
DENV-specific T cell response to Dengue Virus Infection. T cell epitope-based vaccines activate CD4^+^ and CD8^+^ T cells through antigen-presenting cells (APCs) that present T cell epitopes via MHC class II or I, respectively. CD4^+^ T cells function as Th1 cells producing IFN-γ and IL-2 to activate CD8^+^ T cells and macrophages, as Tfh cells promoting the production of high-affinity antibodies and memory B cells, and as Treg cells secreting IL-10 and TGF-β to suppress immune overactivation, while CD8^+^ T cells induce apoptosis of infected cells via perforin and granzyme and suppress viral replication through IFN-γ and TNF-α, ultimately establishing strong and long-lasting antiviral immunity (Figure Created in BioRender. YiFei Sun. (2025) https://BioRender.com/illustrations/688cba2d982a0f79bf1233b5).

Previous research found that most IFN-γ producing CD8+ T cells were memory cells by determining the CD8+ T cell responses using a peptide mega-pool containing 268 DENV-derived epitopes, with the involvement of CD45RA^−^CCR7^−^ (Tem) or CD45RA^+^CCR7^−^ (Temra). Isolated IFN-γ-producing DENV-specific CD8+ Tem and Temra cells from healthy donors who had secondary DENV infections showed increased expression of genes linked to activation, co-stimulation, and effector functions. These findings suggest that CD8^+^ T cells specific to DENV may stay in an activated state in people who have experienced more than one DENV infection [47,48]. This sustained activation could contribute to enhanced protective immunity upon subsequent exposures, providing a rapid and effective cellular response. DENV-specific CD4+ T cells exhibit broad targeting capabilities, recognizing epitopes derived from both structural proteins, such as the capsid and envelope, and secreted non-structural proteins like NS1 [49]. Some CD4+ T cell subsets primarily function by producing and secreting key cytokines (e.g., IFN-γ, TNF-α, IL-2) that actively promote antiviral activity and orchestrate other immune cells. Other CD4+ T cells operate as T follicular helper (Tfh) cells, characterized by their expression of CXCR5 and PD-1, and are crucial for providing direct help to B cells, thereby facilitating the generation of high-quality, neutralizing antibodies. Furthermore, studies have identified cytotoxic CD4+ T cells that express perforin and granzyme B, adding another critical layer of defense by directly eliminating infected cells [49,50,51,52] (Figure 1).

A comprehensive understanding of the T cell-mediated immune mechanism during DENV infection would be critical in improving dengue vaccine design. A successful vaccine should possess the capability of inducing not only robust T-cell responses but also strong and durable neutralizing antibodies to provide cross-protection against all DENV serotypes. Emerging data increasingly underscore the pivotal importance of T cell responses in vaccine efficacy, particularly those targeting conserved epitopes across the DENV serotypes. Notably, cross-reactive CD8+ T cells have been found to offer substantial protection against DENV, even in the presence of disease-enhancing antibodies. This compelling observation unequivocally highlights the urgent need to prioritize the inclusion of conserved epitopes in vaccine design, as they can confer broad and protective immunity that may circumvent the challenges of ADE [53,54]. Additionally, several studies suggest that the strength and breadth of DENV-specific T cell responses could significantly influence the risk of severe dengue, especially for seronegative individuals receiving certain vaccines [55,56]. The observed variation in T cell responses among individuals may therefore be a crucial factor in determining the severity of the disease in these vulnerable populations, emphasizing the importance of understanding, and optimizing T cell-mediated immunity for effective vaccine development.

Moreover, recent work shows that dengue virus (DENV)–specific memory T cells can recognize conserved Zika virus (ZIKV) epitopes [57]. Up to 70% of DENV-immune donors display robust CD4+ and CD8+ T cell responses to ZIKV NS3 peptides ex vivo (reaching 100% after in vitro expansion), whereas cross-reactivity toward ZIKV capsid is negligible [58,59,60,61]. These cross-reactive T cells produce interferon-γ, tumor-necrosis factor-α and CD107a and can lyse ZIKV-infected cells; nine cross-reactive epitopes—mostly in NS3—have been identified [59,60,61,62,63]. Complementary HLA-transgenic mouse studies demonstrate that prior DENV infection narrows the anti-ZIKV CD8+ T cell response to a set of cross-reactive epitopes, and that immunization with ZIKV-specific or DENV/ZIKV cross-reactive peptides protects against ZIKV challenge; depletion of CD8+ T cells abrogates this protection [61,62,63,64,65,66,67,68]. Together, these findings suggest that pre-existing DENV-specific T cells can contribute to cross-reactive and potentially protective immunity against ZIKV, particularly by targeting conserved non-structural proteins. Overall, a deeper understanding of cross-reactive T-cell biology will inform rational vaccine design and improve efficacy in flavivirus-endemic regions.

## 4. Development of T-Cell Epitope-Based Vaccines for Dengue

Traditional vaccines, typically engineered from either live-attenuated or inactivated viruses, are designed to elicit a comprehensive immune response that encompasses both humoral (antibody-mediated) and cellular (T-cell mediated) immunity. By stimulating both arms of the adaptive immune system, these vaccines effectively help to build a robust and long-lasting immune memory. Immune memory is crucial for providing long-term protection against future infections, enabling a rapid and potent response upon re-exposure to the pathogen. Beyond the critical role of antibody responses, T cells play an indispensable and multifaceted role in protecting the body during viral infections. The specific T cell immune responses to a given antigen can vary significantly from person to person. This inter-individual variation primarily depends on two main host-related factors. First, it hinges on the ability of a specific antigen-derived peptide to bind effectively to an individual’s Human Leukocyte Antigen (HLA) class I or class II molecules. These HLA molecules are essential for presenting processed viral peptides to T cells on the surface of antigen-presenting cells. Only peptides that can successfully bind to these HLA molecules will be presented in a manner that can be recognized by T cells. Second, the variation depends on whether the presented peptide–HLA complex is subsequently recognized by the individual’s T cell receptors (TCRs) [69,70]. This recognition is a highly specific molecular interaction mediated by the TCRs expressed on the surface of T cells. When a TCR successfully binds to its cognate peptide–HLA complex, it triggers a targeted and highly specific immune response, leading to T cell activation, proliferation, and effector functions [69,71].

**Figure 2:**
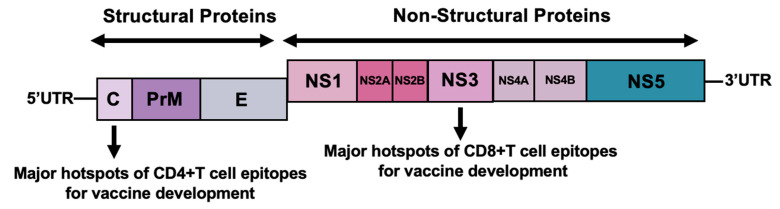
Genomic structure of dengue virus and major hotspots of T cell epitope. Most CD8+ T cell responses are triggered by epitopes found in the NS3 protein. In contrast, CD4+ T cells mainly recognize the capsid. This region serves as the primary immunodominant site for CD4+ T cell activation.

An ideal vaccine should mimic the natural immune response of a natural infection, as observed in clinical and field studies. In this context, the vaccine should not only induce robust antibody responses but also induce both antibody-based and T cell-based memory for a balanced protective response. T-cell epitope-based vaccines represent a strategic approach in comparison to traditional vaccines that primarily depend on inducing high titers of neutralizing antibodies [72,73]. The modality of T-cell-based vaccines relies on utilizing small, select fragments of DENV peptide sequences (Figure 2). These T-cell peptides are selected for the ability to be recognized and responded to by T cells, often targeting highly conserved regions of the viral proteome. This targeted methodology encourages the generation of a strong and highly specific immune response that is predominantly driven by T cells. A major advantage of this approach lies in its potential to offer effective protection in scenarios where traditional antibody-centric vaccines may prove inadequate or even detrimental. T-cell epitope-based vaccines may provide superior results in terms of both safety and effectiveness, as they are designed to minimize the risk of undesirable immune reactions, such as ADE, which has been a significant concern with some conventional dengue vaccine candidates [74,75,76,77]. By enhancing T-cell response, including that of T-helper cells, to hamper or reduce the ADE effect, the T-cell epitope vaccines may help prevent problems observed in some conventional vaccine responses, particularly in developing a multivalent vaccine against dengue.

By strategically triggering both CD8+ and CD4+ T cells, these vaccines foster a strong, coordinated, and multifaceted immune response to enable cross-protective immunity across all DENV serotypes. To further enhance the breadth and potency of this response, vaccine developers frequently incorporate multiple epitopes into their vaccine designs. This multi-epitope approach helps the immune system recognize a broader range of viral variants, which is particularly advantageous for viruses like DENV that exhibit significant genetic diversity across their serotypes and may lower the ADE risk against other secondary flavivirus infection. In the specific context of dengue virus, this comprehensive strategy significantly increases the chances of achieving broad protection against all four serotypes [74,78], a key goal for any successful dengue vaccine. The inherent flexibility in designing T-cell epitope-based vaccines allows for the inclusion of selective peptides that are crucial for mitigating the risk of adverse immune responses [73,79]. Traditional vaccines often face the challenge in providing comprehensive protection across all serotypes and, in some cases, may even exacerbate the disease during a secondary infection due to ADE [39,80,81]. In contrast, T-cell epitope-based vaccines are designed to direct the immune response towards conserved epitopes that are shared across different serotypes of the dengue virus. This strategic focus helps to reduce the risk of serotype specific immune responses that could otherwise exacerbate disease severity during subsequent infections [55,82]. Conserved regions of the dengue virus are usually less immunogenic and often buried within the viral structure, making them inaccessible to antibodies or immune cell surface markers [83,84,85,86]. However, targeting these stable and mutation-resistant regions through T cell–based vaccine strategies offer distinct advantages. Such vaccines can elicit robust, potent, and broad cellular immune responses that provide long-lasting protection across dengue virus serotypes and variants [87,88]. By focusing on conserved epitopes, T cell–based dengue vaccines may maintain effectiveness even as new variants emerge and can offer protection in situations where traditional antibody-focused vaccines are less effective.

Comprehensive research on human T cell epitopes in DENV infection has revealed two major patterns of immune targeting that are critical for rational vaccine design. The first involves the identification of conserved CD8^+^ T cell epitopes predominantly located within non-structural (NS) proteins such as NS3 and NS5, which play essential roles in viral replication and are relatively conserved across all four DENV serotypes (Table 2). These conserved NS5 epitopes cluster in functionally constrained regions (priming loop/motif F or structural interfaces), explaining why they remain cross-serotype and attractive for universal T-cell vaccines. One research group prioritized many of the above as “top 55” conserved epitopes from human data; several (e.g., DTTPFGQQR/TPFGQQRVF/KTWAYHGSY/GPGHEEPIPM) are explicitly called out and structurally mapped, while KPGTSGSPI spans the NS3 catalytic triad (S135) and is strongly conserved [49,53,89,90]. Also, these conserved regions include shared epitopes such as “MYFHRRDLR” and “LPAIVREAI,” making them strong candidates for eliciting broad and durable cross-serotype immunity. Other important CD8^+^ T cell targets include epitopes in the capsid and NS4B proteins, all recognized by cytotoxic T lymphocytes [88,91,92,93,94]. In contrast, CD4^+^ T cell and serotype-specific responses tend to focus on more variable structural proteins, particularly the envelope (E) protein, as well as NS3 and NS4B, where subtle amino acid variations—for instance, QEGAMHTAL versus QEGAMHSAL in the E protein—distinguish serotypes [53,95,96] (Table 3). The capsid protein also serves as a major immunodominant site for CD4^+^ T cell activation, orchestrating the overall immune response [73,97]. These observations suggest that an optimal vaccine strategy should combine conserved epitopes from NS3 and NS5 to achieve broad cross-serotype protection, while incorporating selected serotype-specific epitopes from E, NS4A, and NS4B to finetune and balance immune responses against each DENV serotype [73,97,98]. A compelling analysis of experimentally determined DENV T-cell epitopes identified 14 epitopes that were found (based on the latest available data) to be highly conserved across all four serotypes (Table 4). This significant finding strongly suggests that these specific, highly conserved epitopes could play a crucial role in the development of a universal T-cell vaccine capable of targeting and providing protection against various DENV serotypes [99,100].

Furthermore, studies of flaviviruses show that T cell responses follow a common pattern. Non-structural proteins, especially NS3 and NS5, are the main targets of cellular immunity [57]. Within NS3, the protease region tends to be virus-specific, while the helicase region is more conserved. These conserved regions trigger cross-reactive T cell responses between DENV and ZIKV [57,60]. Such responses can persist for years and contribute to long-term immune memory [70,105]. In people who have been exposed to dengue, strong T cell recognition occurs against ZIKV NS3 peptides [97]. In contrast, responses to ZIKV capsid peptides are weak [68,106]. This shows that cross-reactive T cells mainly recognize non-structural proteins [57,96]. Similar findings have been reported for Japanese encephalitis virus (JEV). Most T cell activity targets NS3, NS4, and NS5 proteins. These responses also show broad cross-reactivity between related viruses [107,108,109]. However, in people who have not been infected with dengue, certain JEV specific epitopes—such as the P34 peptide from the E protein—are unique and act as virus specific controls [107]. Analyses of dengue epitopes confirm that conserved regions in NS3 and NS5 are strong vaccine targets [53,110,111]. These areas are structurally stable and less likely to mutate, making them reliable components for vaccine design [107,112]. Thus, these results point to practical strategies for vaccine development. Thus, these results point to practical strategies for vaccine development, suggesting that inclusion of conserved NS3-helicase and NS5 epitopes may provide broader protection. It is suggested that the inclusion of selected NS3-protease and JEV-specific E epitopes may serve as a rational strategy to preserve viral specificity and minimize unwanted cross-reactivity [53,113,114]. Focusing on stable regions may help reduce escape mutations. Designing T cell-based vaccines around non-structural proteins can also lower the risk of ADE and improve safety [115,116,117] (Table 5).

Immunoinformatic methods play an increasingly important role in the rational design of T-cell epitopes for dengue vaccines. One of the primary applications of these sophisticated computational tools involves mapping known T-cell epitopes onto the protein sequences derived from all four DENV serotypes. This systematic process is crucial for identifying conserved epitopes that possess the capacity to induce strong and cross-reactive immune responses across different serotypes [53]. By precisely targeting these conserved epitopes, vaccines can potentially provide broader and more robust protection against dengue infection, overcoming the limitations of serotype-specific immunity. For instance, a detailed analysis revealed that 29 out of 167 experimentally identified T cell epitopes were not conserved across the vaccine antigens [119]. This finding highlights a critical challenge in the design of effective vaccines: the judicious selection of epitopes that can not only elicit a strong and protective immune response but also minimize the risk of generating escape variants. Escape variants are viral strains that have mutated in a way that allows them to evade the vaccine-induced immune response, thereby reducing vaccine effectiveness. Therefore, careful, and strategic selection of epitopes is crucial to ensure the vaccine’s long-term effectiveness and to prevent potential weaknesses or gaps in the immune response [119,120].

Moreover, the accurate prediction of effective T-cell epitopes is a vital and complex step in the development of T-cell epitope-based vaccines. One groundbreaking study introduced a novel method that leverages insights from idiotope-driven T-cell and B-cell collaboration [121]. The method is derived from Jerne’s immune network theory [122], which posits that immune cells can recognize and regulate each other through shared molecular features, forming a complex interconnected network. Building on this theoretical framework, the B-cell receptor (BCR) sequences were used to identify segments that structurally mimic pathogen proteins. A significant proportion of the identified epitopes exhibited characteristics indicative of strong immunogenicity, including somatic hypermutations, robust MHC binding capabilities, and the ability to effectively stimulate T cells. The results suggest the potential of using T-cell epitope predictive algorithm and artificial intelligence in designing effective DENV vaccines by identifying highly immunogenic T-cell targets that can elicit potent and protective immune responses [123].

## 5. Challenges of Dengue Vaccine Development

Despite the significant progress and promising avenues in dengue vaccine development, particularly with T-cell epitope-based vaccine development strategies, substantial challenges persist that continue to hamper the development of a universally safe and effective vaccine. In this context, ADE raises critical questions about DENV vaccine safety, especially when considering its administration to individuals who have not been previously exposed to the virus (seronegative individuals) [124]. Additionally, the structural similarities among various flaviviruses, including Zika virus, Japanese encephalitis virus, and yellow fever virus, further complicate vaccine development. In addition to pre-existing immunity to DENV, cross-reactive antibodies generated against a flavivirus can pose a significant risk for ADE when an individual is subsequently exposed to DENV. Here, the pre-existing immunity to other flaviviruses, whether from natural infection or vaccination, can potentially modulate responses to DENV vaccines or even enhance infection with other viruses. This complex immunological landscape underscores the critical importance of rigorously evaluating vaccine safety in populations with varying histories of flavivirus exposure and maintaining robust, long-term surveillance following vaccination. This is particularly crucial given observations of an increased risk of severe dengue occurring 18–36 months after vaccination with certain candidates, highlighting the need for careful long-term follow-up [125,126,127].

Evaluating the quality and protective capacity of the immune response elicited by dengue vaccines also remains a considerable challenge. The plaque reduction neutralization test (PRNT) is widely employed as the gold standard assay to assess neutralizing antibodies. However, the PRNT suffers from significant variability between laboratories, making it difficult to compare results consistently across different studies. More importantly, it exhibits an imperfect correlation with actual clinical protection against dengue infection. This discrepancy underscores a clear and urgent need for the development of more reliable and standardized assays, as well as the identification of robust correlates of protection that can accurately predict vaccine efficacy in humans [128,129,130,131,132,133,134,135]. Without precise and consistent measures of immune protection and understanding of the proxy of protection against dengue and other flaviviruses, optimizing vaccine design and assessing the true clinical benefit of new candidates will be challenging.

Furthermore, the efficacy of a vaccine varies considerably due to differences in the adjuvants used in its formulation. In this context, the variation in efficacy, depending on the adjuvant, underscores the urgent need for more extensive research to optimize vaccine formulations [38]. The final goal of this optimization will be to improve on the magnitude, breadth, and durability of immune responses, thereby enhancing vaccine effectiveness across diverse global populations, which exhibit significant genetic and immunological variability. Addressing these multifaceted challenges—ADE, cross-reactivity, accurate immune assessment, and adjuvant optimization—is paramount for the successful development and deployment of a safe and effective dengue vaccine.

## 6. Vaccine Design and Methods

T-cell epitope-based vaccines for dengue are poised to offer a profoundly promising and transformative new strategy in the ongoing global effort to combat this debilitating disease. One of the central focuses of current and future research is on inducing exceptionally strong, broad, and cross-reactive CD8+ T-cell responses [73]. These responses are designed to target all four DENV serotypes, thereby aiming to provide comprehensive and durable effective immunity. This approach is particularly critical given the tetravalent nature of DENV [89] and the well-documented risk of severe complications arising from secondary infections with a different serotype. By strategically addressing this fundamental immunological challenge, T-cell epitope-based vaccines aim to provide broader and more durable protection, significantly reducing the burden of severe dengue.

Future strategies for improving dengue vaccines will increasingly involve the sophisticated integration of advanced immunoinformatic methods. These cutting-edge computational tools have already demonstrated considerable success in the rational design of multi-epitope vaccines against a diverse range of pathogens [55,78,79,136]. In the context of dengue, these methods can be precisely applied to identify, predict, and optimize T-cell epitopes, thereby potentially enhancing the overall immunogenicity and protective capacity of these novel vaccines. This data-driven approach allows for the selection of the most potent and broadly reactive epitopes, maximizing the vaccine’s effectiveness. In addition to this, innovative sequential vaccination strategies could be employed. For instance, by using epitope-decreasing antigens in a phased approach, this strategy may help to strengthen cross-reactivity and improve both T and B cell responses in a controlled and optimized manner [137]. This could involve priming the immune system with a broader set of epitopes and then boosting with a more refined set, or vice versa, to guide the immune response towards desired protective pathways.

In addition to the vaccine antigen design, next generation drug delivery systems (DDS) are rapidly emerging as a highly promising and versatile tool to significantly improve vaccine effectiveness. Nanoparticle delivery systems offer numerous advantages, including enhanced antigen presentation to immune cells, improved stability of vaccine components, and more efficient delivery to the appropriate immunological compartments, all of which lead to a stronger and more targeted immune response. One notable example is the PepGNP-Dengue vaccine, which utilizes gold nanoparticles as a delivery platform for dengue peptide antigens [56,138]. It contains dengue CD8+ T cell epitopes attached to gold nanoparticles. In a phase 1 trial, this vaccine produced strong and diverse T cell responses. No significant anti-DENV antibodies were detected. In contrast, the low-dose group showed clear increases in CD137+ CD69+ CD8+ T cells and memory subsets, including Tcm and TemRA cells. Researchers also found more CXCR3+ Tcm cells, suggesting possible formation of skin resident memory T cells. These results show that the vaccine works through a cell-mediated mechanism driven by cytotoxic CD8+ T cells activated by antigen-presenting cells in the skin. It does not rely on CD4+ T cell or B cell-mediated antibody responses. This design offers a safe way to induce virus-specific cellular immunity while lowering the risk of ADE [138]. This vaccine candidate has already shown promising safety and immunogenicity profiles in early clinical studies, indicating its substantial potential as a viable solution for dengue prevention. These positive results strongly suggest that nanoparticle-based platforms could be crucial for the future development of highly effective dengue vaccines [138], offering a flexible and potent means to deliver T cell epitopes and other antigens.

Another example is an mRNA vaccine that uses lipid nanoparticles. It encodes fragments from the NS3, NS4B, and NS5 proteins [111]. This vaccine protected HLA class I transgenic mice without producing neutralizing antibodies. It shows that a single mRNA molecule can deliver many T cell epitopes at once. Across these vaccine designs, several shared features have emerged. Short linkers, such as AAY for cytotoxic T cells and GPGPG or EAAK for helper T cells, are used to connect epitopes. These linkers improve how the immune system processes the peptides [78]. The chosen epitopes often come from conserved regions of NS3 and NS5 to ensure broad coverage across different HLA types [53]. The delivery method also depends on the goal. mRNA and DNA vaccines are better for antigens that need MHC-I processing, while nanoparticles can present peptides directly [124,139,140]. Some vaccines include LAMP-1 signals to help MHC-II presentation or ubiquitin tags to improve antigen trimming [141,142,143]. At the same time, epitopes linked to ADE are removed or replaced for safety [144,145,146]. These advances show that T cell-based dengue vaccines are moving beyond theory. Some designs are already in clinical trials, while others have shown protection in preclinical studies. In summary, they provide a practical path toward safer and more effective dengue vaccines. As the global challenge posed by dengue continues to grow, innovative and multifaceted strategies are essential to developing an effective vaccine.

## 7. Future Perspectives

Future dengue vaccines are expected to integrate rational epitope design with advanced delivery systems to achieve broad, durable, and safe protection. Epitope selection focuses on conserved regions within NS3 and NS5 that are shared across all four dengue serotypes, such as DTTPFGQQR, KPGTSGSPI, and MYFHRRDLRL. Structural epitopes associated with ADE are excluded to enhance safety. This design strategy aims to elicit strong CD8+ T cell responses while minimizing risks from non-neutralizing antibodies.

The choice of delivery platform is aligned with the immunological goal mRNA and DNA vaccines are used for antigens that require intracellular processing, whereas nanoparticle and peptide platforms enable direct antigen presentation. Signal tags such as LAMP-1 or ubiquitin further improve antigen routing and presentation efficiency. Prime–boost vaccination regimens enhance the magnitude and diversity of T cell responses. The peptide–gold nanoparticle vaccine PepGNP-Dengue demonstrates robust CD8+ T cell activation without antibody induction, supporting its favorable safety profile. Larger clinical studies are warranted to evaluate its long-term efficacy, durability, and optimal delivery route. Future assessments consider both antibody quality and T cell functionality to define accurate correlates of protection. Vaccine design also considers regional HLA diversity (such as HLA-A*24:02, B*07, and B*35) to ensure broad global applicability. Continued optimization of epitope selection, delivery efficiency, and population-based HLA coverage remains critical for achieving a universal dengue vaccine.

## Figures and Tables

**Table 1:** Dengue vaccines in the development pipeline.

Name	Strategy	Clinical Trial Phase	Serotypes	Adjuvant	Reference
Dengvaxia	YFV 17D backbone with the PrM and E proteins of DENV	Licensed	Tetravalent	No	[19,20]
TV003/TV005	One chimeric virus with three attenuated viruses	In vivo (phase IIIB)	Tetravalent	No	[21,22,23]
TAK-003	Live-attenuated. DENV-2 virus genetic backbone for all four serotypes	In vivo (phase III)	Tetravalent	No	[24,25,26,27,28]
TDEN	DENV-1 (45AZ5), DENV-2 (S16803), DENV-3 (CH53489) and DENV-4 (341750)	In vivo (phase I–II)	Tetravalent	No	[29,30]
V180	Recombinant prM and a truncated form of the E protein (covering ~80%) from each of the four DENV serotypes.	In vivo (phase I)	Tetravalent	Yes	[31,32]
TVDV	DNA vaccine based on prM and E protein coding sequences cloned in VR1012 plasmid and co-administered with VAXFECTIN as an adjuvant	In vivo (animal and phase I)	Tetravalent	Yes	[33,34,35]
DSV4	Virus-like particle vaccine designed to present EDIII of all four DENV serotypes	In vivo (animal)	Tetravalent	No	[36]
E80 mRNA vaccine	A modified mRNA-lipid nanoparticle (mRNA-LNP) formulation encoding the DENV E80 protein.	In vivo (animal)	Tetravalent	No	[37]

**Table 2:** Conserved T-cell epitopes shared across DENV1–4 *.

Protein	Epitope (aa)	Conservation (Serotypes)	Class/ Example HLA(s)	Evidence	Reference
NS5	DTTPFGQQR	1–4	Class I; e.g., B*35/B*07 families	Human datasets & structure mapping	[53]
NS5	TPFGQQRVF	1–4	Class I; A*02/B*35 families	Human datasets & structure mapping	[53]
NS5	KTWAYHGSY	1–4	Class I; multiple predicted binders	Human datasets & in silico binders	[53]
NS5	GPGHEEPIPM	1–4	Class I; multiple	Human datasets; interfaces in NS5	[53]
NS5	WSIHAHHQW	1–4	Class I; multiple	Human datasets; priming loop/motif F	[53]
NS5	TWSIHAHHQW	1–4	Class I; multiple	Human datasets; priming loop	[53]
NS5	PTSRTTWSIH	1–4	Class I; multiple	Human datasets; priming loop	[53]
NS5	CVYNMMGKREKKLGE	1–4	Class I/II (overlapping)	Human datasets; motif F proximity	[53]
NS5	KVRKDIPQW	1–4	Class I; multiple	Human datasets; inter-dimer interface	[53]
NS3	KPGTSGSPI	1–4	Class I; multiple	Human datasets; includes protease S135	[53]
NS3	LPAIVREAI	1–4	Class I; B*07:02	Human (Sri Lanka cohort), vaccinology	[99]
NS5 (motif)	MYFHRRDLRL	1–4	Class I; multiple (predicted)	In silico cross-serotype NS5 motif	[90]

* Representative but not exhaustive.

**Table 3:** Serotype specific or serotype biased T-cell epitopes.

Protein	Epitope (aa)	Serotype Specificity/Biased(s)	Class/HLA	Evidence	Reference
E	QEGAMHTAL vs. QEGAMHSAL	DENV1–3 vs. DENV4 (binary variant)	Class I (various)	Reported conservative (binary E variants)	[53]
E (DENV3)	TPTWNRKEL	DENV3 immunogenic	Class I; B*07:02	Human ex vivo ELISpot	[95]
E (DENV3)	RKELLVTFKNAHAKK	DENV3 immunogenic	Class II; DRB1*15:01 (DR2)	Human ex vivo ELISpot	[95]
NS3 (DENV3 core)	KLNDWDFVV (399–407)	DENV3 core; DENV2/4 altered ligands	Class I; A*02:01	Human & Tg mice; variant analysis	[95]
NS3	KPRWLDARI vs. RPKWLDARV	DENV2 vs. DENV3	Class I; B*07 family	HLA-B*07:02 transgenic model	[101]
NS5	TPRMCTREEF vs. KPRLCTREEF	DENV2 vs. DENV3	Class I; B*07 family	HLA-B*07:02 transgenic model	[101]
NS4A	WYAQIQPHWI (96–105)	DENV2	Class I;A*24:02	HLA-A*24:02 transgenic model	[102]
NS4B	HPASAWTLYA/RPASAWTLYA	DENV2/1/4 vs. DENV1/3	Class I; B*35/B*07	HLA-B*07:02 transgenic model	[101,103]
NS3/NS4B	APTRVVAAEM/APTRVVASEM	DENV2/3/4 vs. DENV1	Class I; B*07:02	Human, vaccine & cross-flavi data	[104]

* Representative but not exhaustive.

**Table 4:** Highly conserved epitopes that could be applied to the development of multiple epitope-based vaccine candidates.

Epitope Sequence	Position
AMTDTTPF	NS5 protein
CVYNMMGKREK	NS5 protein
FTNMEAQL	NS5 protein
LMYFHRRDLRL	NS5 protein
MYFHRRDLRL	NS5 protein
LMYFHRRDL	NS5 protein
WYMWLGAR	NS5 protein
LEFEALGF	NS5 protein
YFHRRDLR	NS5 protein
DTAGWDTR	NS5 protein
TFTNMEAQL	NS5 protein
VPTSRTTWSI	NS5 protein
MYFHRRDLRL	NS5 protein
LHKLGYIL	NS5 protein

**Table 5:** Cross-virus T-Cell Epitope Comparison (DENV/ZIKV/JEV).

Peptide Sequence	Virus/Protein & Position	Domain (Protease/Helicase/Polymerase/E, etc.)	HLA Restriction(s)	Evidence/Assay (Human Preferred)	Cross-reactivity/Specificity Notes	Reference(s)
“KLNDWDFVV”	DENV NS3 helicase (≈position ~400)	Helicase	HLA-A*11:01	ICS/ELISpot in DENV-exposed humans	Recognized in both DENV and ZIKV NS3 homologs (cross-reactive)	[60]
“ELMRRGDLPV”	DENV NS3 protease (≈position ~100)	Protease	HLA-A*02:01	Human T cell mapping (conserved-epitope meta sets)	Likely virus-specific, less cross recognition by ZIKV	[57]
“APTRVVAAEM”	DENV NS5	Polymerase	HLA-A*02:01	CD8 assays in cohorts, IEDB annotation	Highly conserved across DENV, possible partial cross-homology in ZIKV/JEV	[118]
“RVIDPRRCL”	DENV NS3 (helicase region)	Helicase	HLA-B*07:02	Human T cell mapping	Conserved within DENV; homologous positions in ZIKV weaker but possible cross recognition	[57]
“LPAIVREAI”	DENV NS5	Polymerase	HLA-A*02:01	CD8 response in vaccinated or infected donors	Strongly conserved; cross-homology with ZIKV region moderate	[57]
“HYMYLIPGL”	ZIKV (e.g., NS region)	—	HLA-A*24:02	CD8+ epitope mapped in humans or mice	Described as ZIKV-specific (low DENV homology)	[67]
“MTTEDMLQVW”	JEV NS5/NS region	Polymerase/NS5 domain	— (CD8)	Ex vivo ICS in JE-exposed donors	JEV epitope, used to test cross recog with DENV/ZIKV	[107]
“P34 (20-mer)”	JEV E protein	Envelope	CD4 (multiple HLA)	ELISpot in JE vaccinees, especially DENV-seronegative	Largely JEV-specific (low cross in DENV/ZIKV)	[107]

* Representative but not exhaustive.

## Abbreviations

The following abbreviations are used in this manuscript:

DENV	Dengue Virus
ZIKV	Zika virus
JEV	Japanese encephalitis virus
ADE	Antibody-dependent enhancement
DF	Dengue Fever
DHF	Dengue Hemorrhagic fever
DSS	Dengue Shock Syndrome
FcγR	Fcγ receptor
C	Capsid
PrM	Pre-membrane
E	Envelop
NS	Non-structural
mRNA-LNP	mRNA-Lipid Nanoparticle
HLA	Human Leukocyte Antigen
TCRs	T cell Receptors
BCR	B cell Receptor
IFN-γ	Interferon-gamma
TNF-α	Tumor Necrosis Factor-alpha
TGF-β	Transforming Growth Factor-beta
IL-10	Interleukin-10
Tfh	T Follicular Helper
PRNT	The Plaque Reduction Neutralization Test
DDS	Drug Delivery Systems
